# 

**Published:** 1882-10

**Authors:** 


### BOOKS RECEIVED.

**The International Encyclopedia of Surgery: A Systematic Treatise on the Theory and Practice of Surgery by Authors of Various Nations.** Edited by JOHN ASHHURST, Jr., M. D. Illustrated with chromo-lithographs and wood-cuts. In six volumes. Vol. II. New York: William Wood & Co. 1882.

